# Characterization of background noise in capture-based targeted sequencing data

**DOI:** 10.1186/s13059-017-1275-2

**Published:** 2017-07-21

**Authors:** Gahee Park, Joo Kyung Park, Seung-Ho Shin, Hyo-Jeong Jeon, Nayoung K. D. Kim, Yeon Jeong Kim, Hyun-Tae Shin, Eunjin Lee, Kwang Hyuck Lee, Dae-Soon Son, Woong-Yang Park, Donghyun Park

**Affiliations:** 1 Samsung Genome Institute, Samsung Medical Center, Seoul, 06351 Korea; 2 Department of Biomedical Sciences, Seoul National University College of Medicine, Seoul, 03080 Korea; 3 Department of Medicine, Samsung Medical Center, Sungkyunkwan University School of Medicine, Seoul, 06351 Korea; 4 Department of Health Sciences and Technology, Samsung Advanced Institute for Health Sciences & Technology, Sungkyunkwan University, Seoul, 06351 Korea; 5 Department of Molecular Cell Biology, Sungkyunkwan University School of Medicine, Suwon, 16419 Korea

**Keywords:** Next-generation sequencing, Targeted deep sequencing, Substitution rate, Background error, DNA fragmentation, Plasma DNA

## Abstract

**Background:**

Targeted deep sequencing is increasingly used to detect low-allelic fraction variants; it is therefore essential that errors that constitute baseline noise and impose a practical limit on detection are characterized. In the present study, we systematically evaluate the extent to which errors are incurred during specific steps of the capture-based targeted sequencing process.

**Results:**

We removed most sequencing artifacts by filtering out low-quality bases and then analyze the remaining background noise. By recognizing that plasma DNA is naturally fragmented to be of a size comparable to that of mono-nucleosomal DNA, we were able to identify and characterize errors that are specifically associated with acoustic shearing. Two-thirds of C:G > A:T errors and one quarter of C:G > G:C errors were attributed to the oxidation of guanine during acoustic shearing, and this was further validated by comparative experiments conducted under different shearing conditions. The acoustic shearing step also causes A > G and A > T substitutions localized to the end bases of sheared DNA fragments, indicating a probable association of these errors with DNA breakage. Finally, the hybrid selection step contributes to one-third of the remaining C:G > A:T and one-fifth of the C > T errors.

**Conclusions:**

The results of this study provide a comprehensive summary of various errors incurred during targeted deep sequencing, and their underlying causes. This information will be invaluable to drive technical improvements in this sequencing method, and may increase the future usage of targeted deep sequencing methods for low-allelic fraction variant detection.

**Electronic supplementary material:**

The online version of this article (doi:10.1186/s13059-017-1275-2) contains supplementary material, which is available to authorized users.

## Background

Tens of thousands of tumors of varying types have been analyzed using next-generation sequencing (NGS) for systematic variant discovery [1, 2]. This has resulted in the comprehensive characterization of many cancer genomes and we are thus now able to identify genetic alterations that are common to a variety of human tumor types [1, 3]. Having identified these genetic alterations, targeted sequencing techniques can now be used in clinical settings to analyze the cancer-related genomic regions in which they are enriched, thus enabling the cost-effective genomic profiling of somatic variants in tumor specimens [4–6]. However, tumor heterogeneity is common, and often various somatic mutations only occur in a small fraction of cells in a given tumor; hence, there is a growing need for technologies capable of identifying subclonal variants [7, 8]. These variants, particularly at a stage before they become dominant in a given tumor cell population, are likely to be primary factors, contributing to both cancer recurrence and the rapid acquisition of resistance to targeted therapies [9, 10]. Thus, detection of subclonal variants may be essential to optimize therapy outcomes for patients via the selection of timely and appropriate treatment options [9]. Furthermore, it is vital that methods enabling the detection of low-allelic fraction variants be developed and optimized so that, in the clinical setting, standard genetic profiling techniques can be replaced with noninvasive methods to detect and profile tumor variants in plasma and other bodily fluids [11, 12]. Such noninvasive methods are widely applicable, and can be used to monitor minimal residual disease after therapy, to follow disease progression and patient response during therapy, and to identify cancer patients within healthy populations at early and curable stages of disease progression [13–15]. Furthermore, noninvasive methods are not limited to neoplastic diseases, but are also applicable to trauma [16], stroke [17], organ transplantation [18], prenatal screening for fetal aneuploidy [19], etc.

While detection of small DNA subpopulations requires deep sequencing a sufficient number of molecules, a practical limit of detection is also imposed by errors that occur during sample preparation and sequencing [20–22]. A thorough characterization of such errors may facilitate the detection of method-dependent systematic errors, and furthermore allow true variants to be distinguished from these errors. For example, errors caused by Illumina HiSeq sequencer chemistry are relatively well understood, and therefore appropriate data filtering criteria based on this knowledge are routinely applied to generated data to remove them [23]. This filtering includes the removal of parts of reads, or entire reads containing numerous low-quality bases, to minimize downstream analysis artifacts [24].

Nevertheless, it is not clear what fraction of errors are incurred during the sequencing run itself, since technical errors are also likely to be introduced during sample preparation, library preparation, target enrichment, and/or amplification of DNA samples. The fidelity of polymerases routinely used in the construction of sequencing libraries is well characterized [25, 26]; however, it is difficult to quantify the error rate induced by DNA damage during library construction. For example, heat-induced cytosine deamination during PCR thermocycling has been suggested as a possible cause of baseline noise in ion torrent semiconductor sequencing data [27]. Moreover, cytosine deamination occurs not only during experimental procedures such as PCR amplification [27] and formalin fixation [6, 28], but also prior to sample preparation (i.e., intrinsically or biologically) in the original DNA templates [29]. To determine during which step, and to what extent, a given type of error is introduced during sequencing, comparative experiments under different experimental conditions have been recommended, but are rarely performed due to practical reasons [23]. Thus, no systematic analysis of the errors introduced during capture-based targeted deep sequencing has yet been conducted.

However, because our study is not the first to investigate the etiologies of sequencing errors, some of our findings are also supported by previous studies reporting similar sources of artifacts. To compare our findings with the previous studies, we summarize the artifactual changes identified in different studies and key analytical parameters in Additional file 1: Table S1, which is further described in the “Discussion” section. In the present study, we analyzed non-reference alleles in ultra-deep coverage targeted capture sequencing data from both plasma and peripheral blood leukocyte (PBL) DNA samples. From this analysis, we could estimate the rate of sequencing-artifact substitutions incurred during specific steps of the capture-based targeted sequencing process, including DNA fragmentation, hybrid selection, and sequencing run. Based on our results, we recommend the use of mild acoustic shearing for genomic DNA (gDNA) fragmentation to minimize C:G > A:T and C:G > G:C transversion errors. Finally, we estimated the effect of coverage depth on the number of false positives incurred at a constant error rate.

## Results

### Targeted deep sequencing on plasma and peripheral lymphocyte DNA

We previously reported an efficient library construction method that was specifically designed to mitigate the effects of the low amount of sample input DNA available during targeted deep sequencing, via optimized ligation conditions including reaction duration, temperature, and adaptor concentration [30]. Using this method, we profiled paired plasma and PBL DNA samples from 19 human subjects, including two healthy adults and 17 patients with pancreatic cancer. To cost-effectively achieve a mean sequencing depth of ~10,000× (before de-duplication), we designed a pool of RNA baits covering a total of ~499 kb of the human genome, including 83 cancer-related genes (Additional file 2: Table S2). To construct sequencing libraries, 200 ng of DNA was used to construct sequencing libraries for PBL DNA, and 37.3 ng of plasma DNA was used on average (Additional file 2: Table S3). The generated DNA libraries were sequenced using the Illumina HiSeq2500. The average total reads generated from the plasma and PBL DNA samples was 56.3 and 20.0 million reads, respectively. The average read alignment rate was 87.3% for plasma and 93.7% for PBL DNA samples. After excluding PCR duplication from sequencing data, the unique coverage depths for plasma DNA and PBL DNA samples were 1964× (1210 − 3069×) and 1717× (1042 − 2361×) on average, respectively (Additional file 2: Table S3). To exclude the possibility of systemic bias affecting either the library or sequencing data of the different sample types, the allele frequencies of single nucleotide polymorphisms (SNPs) between matched plasma and PBL samples were compared. The results of this analysis showed a strong correlation between SNP allele frequencies in plasma and PBL samples (*R* = 0.9913, *p* value < 0.0001; Additional file 3: Figure S1).

### Errors introduced by the sequencing reaction

**Fig. 1:**
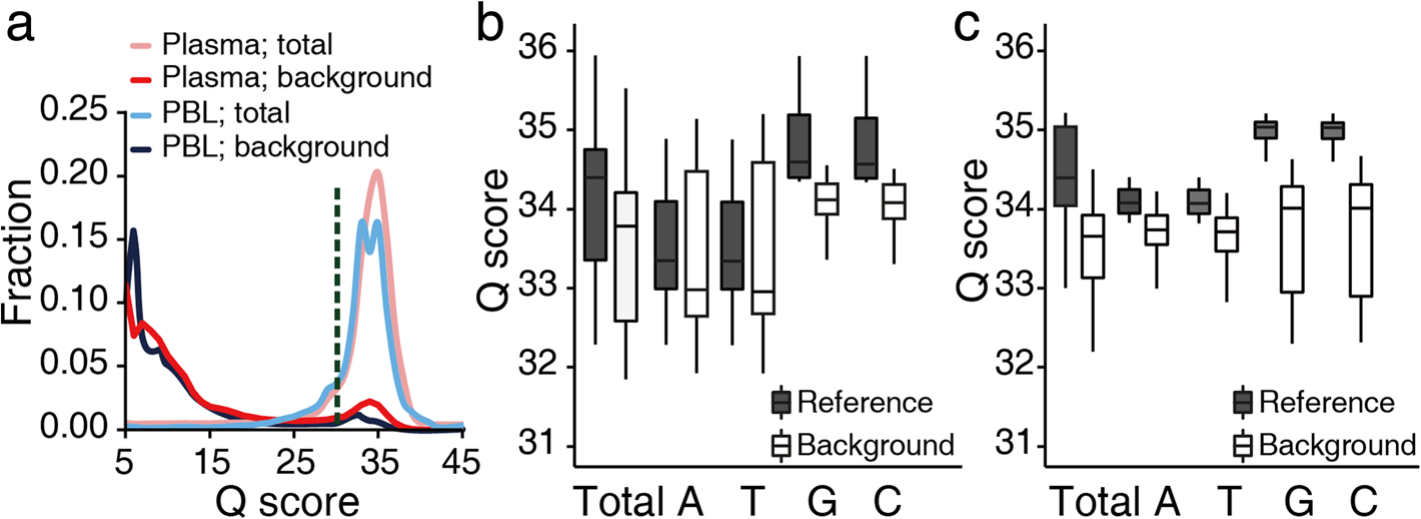
Base call quality in targeted deep sequencing data. **a** The density plot visualizes Phred base quality score distribution of background and total bases. **b**, **c** After the removal of bases with a quality score <30, the average base quality scores (i.e., total and for each of the four nucleotides) was box-plotted for PBL (**b**) and plasma (**c**) DNA samples

After excluding tumor-derived single nucleotide variants (SNVs) and germline SNPs (“Methods”), we investigated the extent to which background error was introduced during the sequencing run by graphing the Phred base quality scores of non-reference background alleles. Our results show that while most background alleles displayed base quality scores of less than 20, a small fraction of background alleles exhibited a quality score distribution indistinguishable from that of the reference alleles (Fig. 1a). Based on the biphasic distribution of the background allele quality scores, we excluded bases with a quality score <30 to remove the majority of sequencing errors for all downstream analyses. In the raw sequencing data, the fraction of bases with a quality score ≥30 was 87 ± 3.3% (mean ± standard deviation (SD)) for PBL and 87 ± 2.5% for plasma DNA samples. After the exclusion of bases with a quality score <30, the overall distribution of base quality scores was observed to be not notably different between background and reference alleles (Fig. 1b, c). This suggests that errors incurred during the sequencing run were largely irrelevant with regards to the background alleles.

Although the overall base quality scores of the background alleles did not significantly differ from those of the reference alleles, the greatest differences between the two groups were observed in the base quality scores for C and G residues, which arose predominantly as a result of A > C and T > G transversions (Additional file 3: Figure S2). This is supported by previous error profile studies of Illumina platforms, which show the frequencies of base substitutions to vary by a factor of between 10- and 11-fold, with A > C and T > G conversions being the most frequent errors observed [31–33]. Similarly, Minoche et al. [24] reported that these substitutions comprised approximately 29 and 27% of all substitutions in generated HiSeq data, respectively. However, A > C and T > G transversions in our data showed the lowest background error rates amongst all the substitution classes (as described below), indicating the limited effect of these errors after excluding low quality bases. Taken together, our data indicate that the majority of background alleles observed after filtering of bases with a quality score <30 were not likely to have occurred due to errors introduced during the sequencing run in either plasma or PBL DNA samples.

Next, we utilized overlapping regions between paired reads 1 and 2 to estimate the extent to which errors incurred during the sequencing run persist after the filtration of low quality bases. If an error occurred prior to the sequencing run, it should be consistently present in both reads 1 and 2, whereas an error incurred during the sequencing run is likely to be inconsistently identified between reads 1 and 2. By analyzing overlapping sequences, we found that the average fraction of inconsistent errors was 18.1% for plasma and 23.6% for PBL DNA samples (Additional file 3: Figure S3). Considering that the overlapping regions represent the end section of reads where errors are more likely to occur during the sequencing run, this result may overestimate the overall fraction of errors incurred during the sequencing run. Nonetheless, our results suggest that the majority of errors with high quality base scores occurred prior to the sequencing run.

### Errors observed after base quality filtration

**Fig. 2:**
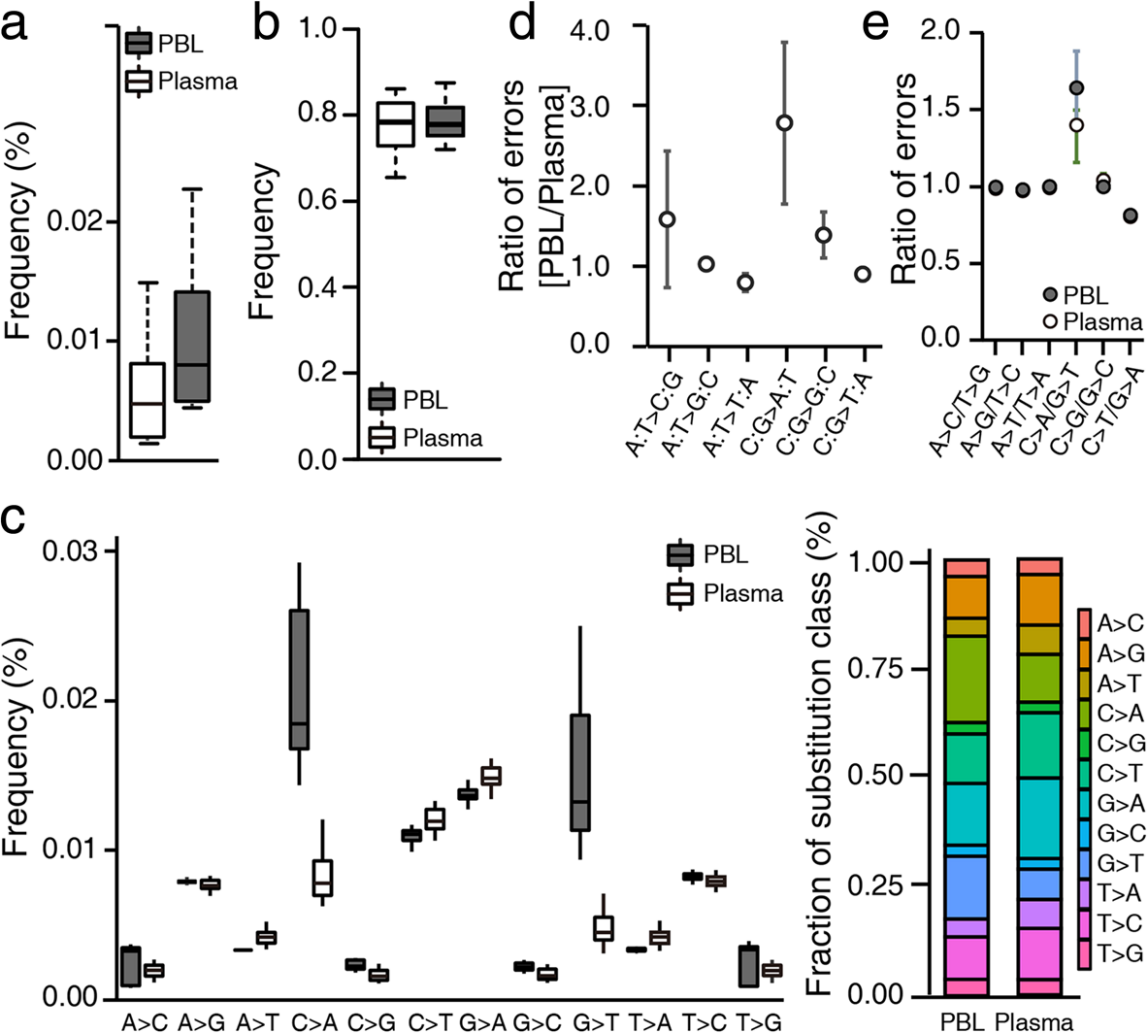
Characterization of background errors in targeted deep sequencing data. **a** The background allele frequencies from plasma and PBL DNA samples were box-plotted (n = 19 for each group). **b** The frequency of error-free positions in each sample was calculated and box-plotted for each group. **c** The distribution of background allele frequencies across all possible 12 substitution classes. The *y-axis* denotes the frequency of each class in the pre-treatment PBL and plasma samples. The relative base substitution frequency is shown in the stacked bar plot on the right side. **d** The ratio of background errors in PBL compared to plasma DNA samples was plotted for each substitution class indicated on the *x-axis.* **e** The ratio of background allele frequencies for reciprocal base substitutions. *Error bars* indicate standard deviation

Based on the distribution of base quality scores, we focused our subsequent analyses on background noise remaining after base quality filtration and thus restricted our analysis of background errors to bases with a quality score ≥30. Initially, we calculated the average allele-specific background rates for the 19 human samples across the entire target regions, and resultantly estimated the overall mean background allele frequency to be 0.007 and 0.008% in plasma and PBL DNA samples, respectively (Fig. 2a). Error-free positions were shown to occur at a frequency of 77.2 ± 1.4% (mean ± SD) for plasma and 78.7 ± 1.0% for PBL DNA samples across the entire target regions (Fig. 2b). Next, we examined errors across all 12 nucleotide substitution classes (Fig. 2c) and combined these data with information regarding the bases located immediately 5′ and 3′ to each mutated base to reveal context dependencies. While the background frequency of each substitution class significantly varied with context, the overall patterns of background frequency variation associated with specific sequence contexts were similar between plasma and PBL DNAs (Additional file 3: Figure S4). Nevertheless, various substitution classes showed obvious differences between plasma and PBL DNA samples (as described in the following sections).

### Errors incurred during the DNA fragmentation step

Since plasma DNA is predominantly fragmented to a length comparable to that of mono-nucleosomal DNA, it is not necessary to shear plasma DNA to enable library construction. Thus, we were able to exploit the naturally fragmented state of plasma DNA to estimate and characterize technical errors introduced during DNA fragmentation. Given that the only difference between the protocols employed for plasma and PBL DNA samples was the fragmentation of DNA by adaptive focused acoustic technology (Covaris), we assumed that any observed PBL-specific errors were due to DNA damage incurred during the fragmentation step. When we performed a statistical test to compare background errors between plasma and PBL DNAs across all substitution classes, we found that C:G > A:T (the colon separates complementary bases written in a 5′ → 3′ direction) and C:G > G:C transversion errors were significantly elevated in PBL compared to plasma DNA samples (Bonferroni adjusted *p* value <10^−4^ for both transversions; Fig. 2d). In fact, C:G > A:T transversions were the most frequent errors identified in PBL DNA samples and, on average, 64% of C:G > A:T transversions occurred seemingly as a result of DNA damage incurred during the fragmentation step. Although the frequency of C:G > G:C transversions was relatively low, it was similarly increased by 39% in PBL compared to plasma DNA samples.

**Fig. 3:**
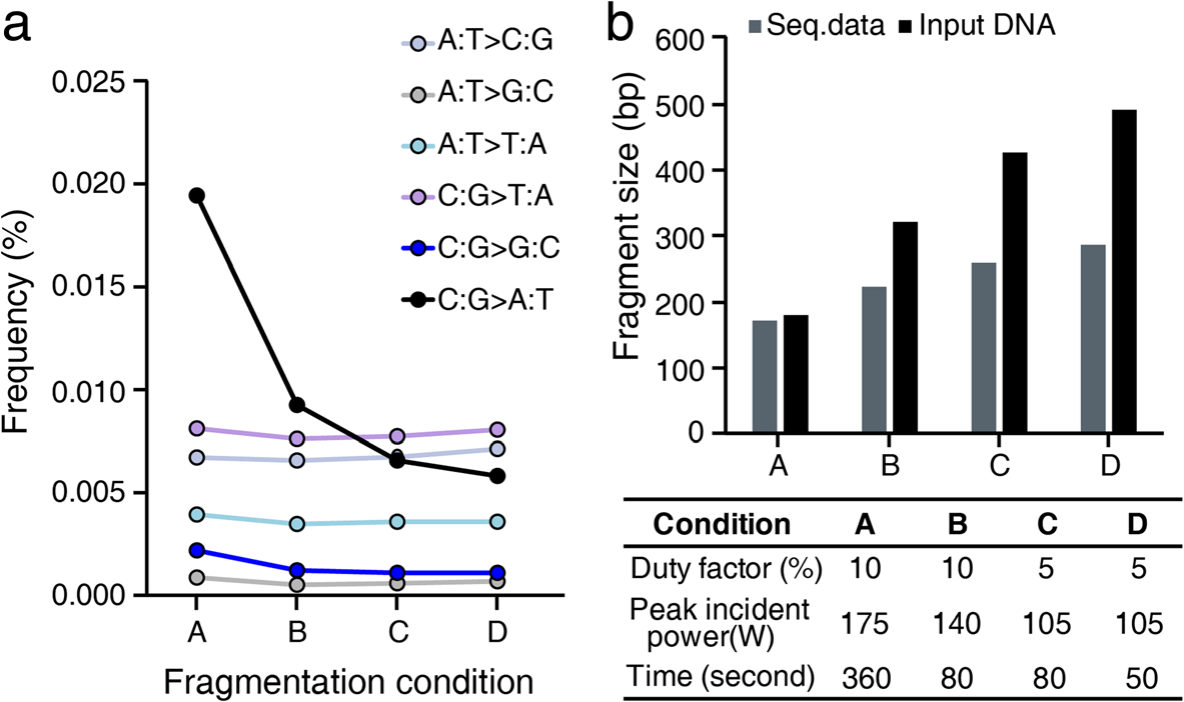
The effect of DNA fragmentation on the background allele frequency. **a** The background error rates were calculated from sequencing data sets generated using genomic input DNA fragmented under various conditions. **b** A detailed description of the conditions used in (**a**). The median DNA fragment sizes of input DNA and inserts of sequencing data were measured. Varied parameters for each condition are indicated in the table

Based on these results, we hypothesized that the elevation of C:G > A:T and C:G > G:C transversion rates in PBL DNA was caused by DNA damage incurred during the acoustic shearing step. To test this hypothesis, we varied the acoustic shearing conditions and thus estimated their effects on DNA damage and induced technical errors. The ultrasonic acoustic energy was lowered by adjusting the intensity and/or shortening the duration of acoustic shearing to lengthen the median fragment size of sheared genomic DNA samples (Fig. 3; Additional file 2: Table S4). When we performed an enzyme-linked immunosorbent assay (ELISA) to measure the formation of 8-oxo-7,8-dihydroguanine (8-oxo-G), which is a typical oxidative base lesion that leads to C:G > A:T errors, we found that lowering the utilized ultrasonic acoustic energy significantly attenuated the 8-oxo-G level (ANOVA *p* value = 6.0 × 10^−7^; Additional file 3: Figure S5a). Furthermore, it also dramatically decreased the rate of C:G > A:T and C:G > G:C transversion in PBL DNA to match those observed in plasma DNA, without affecting the other substitution classes (Fig. 3a). Thus, our data show that the standard DNA fragmentation protocol caused C:G > A:T and C:G > G:C transversions via DNA damage, and that this source of error was alleviated under mild acoustic shearing conditions.

By analyzing the frequencies of these errors with regard to specific sequence context, we found that both C:G > A:T and C:G > G:C transversion errors due to acoustic shearing were most frequently induced in the specific sequence context, NCG:CGN. The fact that this specific sequence context was shared by the two transversion errors implies potential commonality of their underlying mechanisms (as discussed in subsequent sections).

### Errors at the break point of DNA fragments

**Fig. 4:**
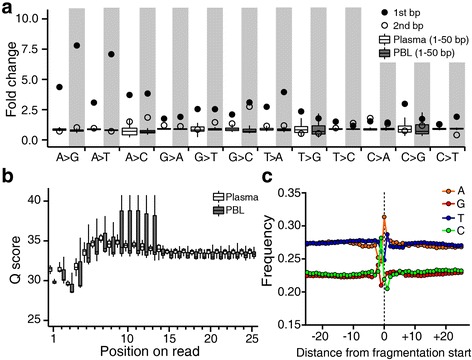
Characterization of the DNA break point. **a** Fold changes in error rates across all substitution classes were compared between PBL and plasma DNA samples. The fold change was calculated by dividing the substitution rate at each position by the average rate of 1 − 50 bp. The distribution of fold changes in 1 − 50 bp was box-plotted. Above the box plots, the observed fold change at the first and the second bases was marked for comparison. For better discrimination between the groups, plasma data are displayed on a *grey background*. **b** The distribution of quality scores of total bases, including low quality bases, is plotted according to the position of reads. **c** Mononucleotide frequencies around the DNA break point. Note: except in (**b**), all analyses of background alleles were performed after filtering of bases with a quality score <30

Next, we considered whether errors introduced during DNA fragmentation might be associated with mechano-chemical breakage of DNA. This association would be expected to produce an accumulation of errors in close proximity to DNA break points, although notably, several other sources of error are likely to confound the analysis of any such accumulation. For example, the Illumina sequencing platform is known to accumulate all types of substitutions in the first 10 bp of reads [33, 34]. Consistent with previous reports, we found base quality scores in the first four consecutive bases of reads to be deceased when low-quality score bases were not excluded from analysis (Fig. 4b). We examined whether background errors with high base quality scores were more likely to arise near the end of a given DNA fragment. From our results, we observed an increase in the rate of errors induced across most substitution classes to occur specifically at the first base of a read (Fig. 4a; Additional file 3: Figure S6a). This dramatic increase in the error rate at the first base was virtually abolished at the second base, as demonstrated via analysis of both PBL and plasma DNA samples (Additional file 3: Figure S6b).

To distinguish error associated with acoustic shearing, we again took advantage of naturally fragmented plasma DNA. We found that the substitution rate of A with either G or T (i.e., A > K) at the first base was significantly elevated in PBL compared to plasma DNA samples (Fig. 4a; Additional file 3: Figure S6). Since the overall A > K substitution rate was not affected by acoustic shearing, we were able to determine that fragmentation-induced A > K substitutions were localized to the end regions of analyzed fragments, suggesting that there may be a strong association between this type of substitution and mechano-chemical breakage of DNA. In contrast, we observed that neither C:G > A:T nor C:G > G:C errors (these being the most common errors induced by acoustic shearing) were significantly elevated in PBL compared to plasma DNA samples, suggesting that such errors are not associated with mechano-chemical breakage of DNA. Although the first-base G > T error rate was observed to be higher in PBL than plasma samples, it was not clear whether the G > T errors were associated with mechano-chemical breakage of DNA because we also observed an overall elevation of these errors in the reads from PBL DNA samples.

To analyze mononucleotide frequency around DNA break points, we aligned generated sequencing reads to the reference human genome to obtain sequencing information pertaining to these regions. We observed mononucleotide frequencies to significantly fluctuate near the break point, and also found that the first base of sequencing reads displayed a significant enrichment in A residues (Fig. 4c; Additional file 3: Figure S7). When we examined the cleavage frequencies of phosphodiester bonds in 16 dinucleotides by calculating their frequencies around DNA break points, we identified CA, TA, and GA to be susceptible to cleavage (Additional file 3: Figures S8 and S9), such that the cleavage rate of phosphodiester bonds diminished according to the following order: CG > CA > TA ~ GA. Thus, our data suggest that acoustic shearing preferentially cleaves the phosphodiester bond at the 5′ side of A nucleotide residues, and furthermore that A > K substitution may be strongly associated with this cleavage.

### Errors at the hybrid selection step

A recent report by Newman et al. [35] indicated that G > T transversions predominated amongst all substitution classes, and occurred much more frequently than reciprocal C > A transversions, in their experiments with NimbleGen SeqCap baits targeting the plus strand. The results of the study suggested that G > T transversions were primarily caused by oxidative damage that occurred during the hybrid capture step [35]. Since we used Agilent SureSelect baits that targeted the minus strand, such DNA damage (if present) might be expected to result in predominant C > A transversions and an imbalance in the ratio of C > A to G > T transversion errors. In fact, we calculated the ratio of C > A to G > T transversions to be 1.67 ± 0.10 (mean ± SD) and 1.44 ± 0.24 in plasma and PBL DNA samples, respectively, indicating the occurrence of guanine residue oxidative damage on the minus strand (Fig. 2e). Compared to the C:G > A:T transversion errors due to acoustic shearing, errors incurred during the hybrid selection step constituted a relatively small fraction. The frequency of C > A transversions (0.0087%) was only 1.7-fold higher than that of G > T transversions (0.0054%), and only slightly greater than the overall substitution rate (0.007%) in plasma DNA, indicating that limited oxidative DNA damage was incurred by the use of Agilent baits (Fig. 2b). The difference in the ratio between plasma and PBL DNAs was likely due to the elevation of C:G > A:T error rate in PBL samples caused by acoustic shearing of genomic DNA. In addition, our data show an imbalance in the ratio of C > T to G > A errors, indicating that cytosine residues were deaminated on the minus strand during the capture hybridization step (Fig. 2e). Although Newman et al. previously reported that C > T substitutions occurred during hybrid selection with NimbleGen SeqCap baits targeting the plus strand, they did not observe the same phenomenon to affect their use of baits to target the minus strand; notably, however, they only analyzed a limited number of data sets [35]. In the present study, our data indicate the occurrence of similar C > T transitions during the hybrid selection step, despite our use of a different hybridization condition comprising minus strand-specific baits. The identified imbalances between complementary substitution classes (i.e., G > T to C > A and C > T to G > A) were consistent between plasma and PBL DNA samples (Fig. 2e).

### The false positive rate

**Fig. 5:**
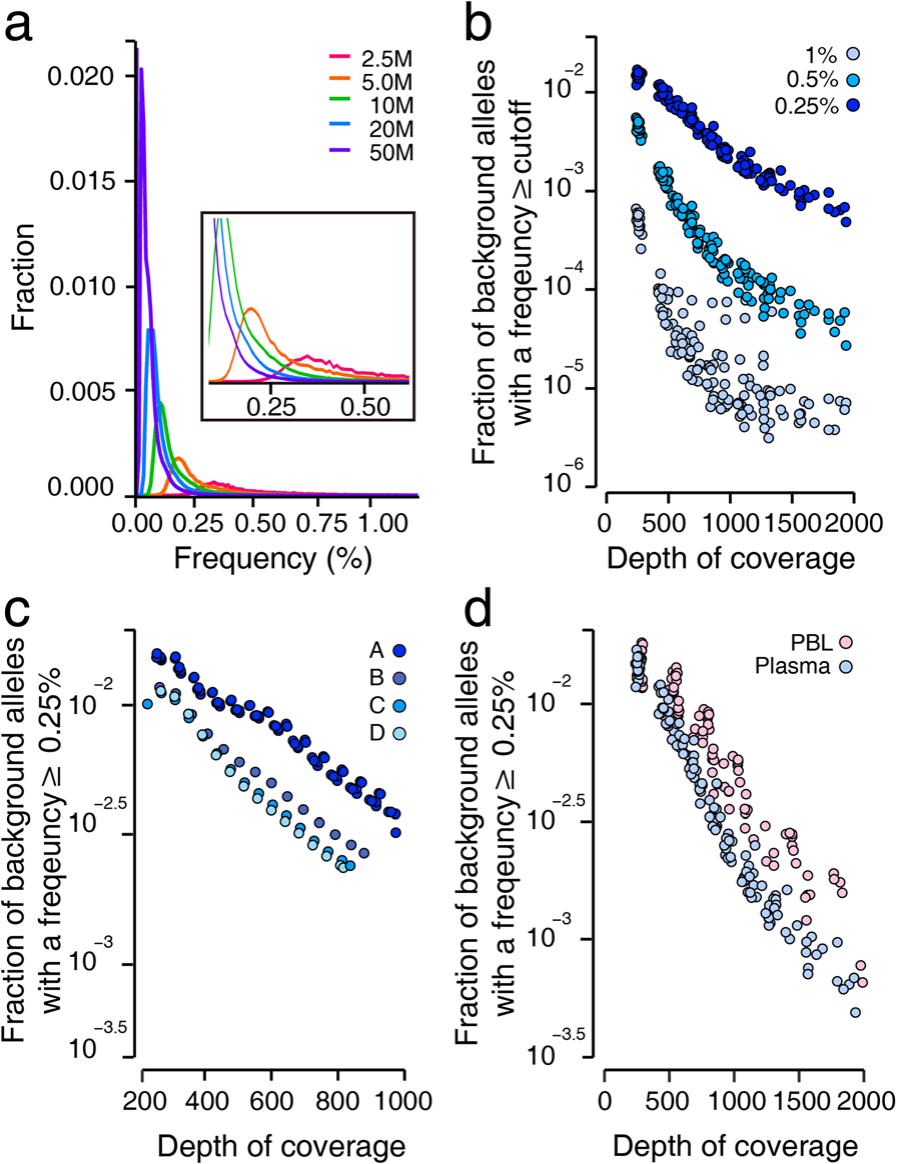
The false positive rate. **a** The error rate of each background allele was calculated and their distribution was plotted, dependent on the total read counts. Data from 19 plasma DNA samples were down-sampled to a designated size of total reads in a range between 2.5 and 50 M. The *x-axis* denotes the frequency of background alleles, and the *y-axis* denotes the fraction of alleles with the designated background rate on the *x-axis*. **b**–**d** The fraction of background alleles present at a frequency greater than a given threshold is plotted against the depth of coverage after de-duplication (*x-axis*). **b** The effect of coverage depth on the false positive rate is shown in the down-sampled data set generated from 19 plasma DNA samples. **c** The effect of each DNA shearing condition on the false positive rate was estimated using the down-sampled data set from Fig. 3. **a–d** indicate the fragmentation conditions, as described in Fig. 3. **d** A comparison of plasma and fragmented PBL DNA samples

Although the mean error rates observed across the entire target regions were relatively low in all analyzed samples, the allelic frequency calculated for each background allele in a given sample varied significantly. This observation is likely to be due in part to significant stochastic variations of rare events, but also to the large variability between allele-specific error rates (Fig. 5a; Additional file 3: Figure S10). Since sample size exerted a profound impact on the magnitude of stochastic variations, the distribution of background allele frequencies changed dependent on the depth of coverage. Our in silico down-sampling analysis showed that a decrease in the depth of coverage improved the fraction of error-free genomic positions but also increased the chance of high-frequency errors (Fig. 4a). For example, the chance of a background allele being present at a frequency greater than 1% was increased by more than an order magnitude as the depth of unique coverage after de-duplication lowered from >1000× to <500×. Thus, at a constant error rate, our data show that low-depth sequencing data were more likely to result in false positives (Fig. 5b).

Next, we estimated how significantly errors caused by DNA shearing influenced the false positive rate, by down-sampling the data from Fig. 3 (which was generated using various DNA shearing conditions) and comparing false positive rates. Compared to the standard condition, the mild shearing condition that attenuated background errors significantly reduced the false positive rate (Fig. 5c). This finding was also supported by a comparison of plasma and PBL samples which showed that, at a given depth of coverage, PBL DNA samples fragmented by the standard acoustic shearing condition were affected by a higher false-positive rate than plasma DNA samples (Fig. 5d).

## Discussion

In the present study, we show that the rates of C:G > A:T and C:G > G:C transversion errors were significantly increased in PBL compared to plasma DNA samples as a result of the acoustic shearing of gDNA. In cells, a plethora of studies have reported the predominance of C:G > A:T and C:G > G:C substitutions induced by DNA exposure to oxidants [36–39]. Relative to the other DNA nucleobases, guanine is more susceptible to the formation of oxidation lesions owing to its low oxidation potential [40, 41]. One such guanine oxidation lesion comprises the formation of 8-oxo-G, which is known to cause G to T transversion substitutions via the dA:8-oxo-G pair [42]. Previously, Costello et al. [43] showed C:G > A:T artifact substitutions to be caused by 8-oxo-G lesions generated during DNA shearing, and also demonstrated that these were reduced by antioxidants. Notably, the C:G > A:T errors analyzed in this study were markedly different from those characterized by the previous study. First, the substitution allele fraction was much smaller in our study compared to that observed previously, such that when the previous study reported C:G > A:T errors to be present at allelic fractions of up to 20%, we found errors with allelic fractions of >1% to be very rare. The previous study analyzed affected samples containing reactive contaminants from the extraction process and showed that the high rates of C:G > A:T errors were not caused by sonication alone, but rather by a combination of sonication and contamination. Second, the predominant sequence-specific context for substitution errors was identified in the previous study to be C**C**G:C**G**G > CAG:CTG (where the target base is bolded), whereas we determined it to be N**C**G:C**G**N > NAG:CTN according to our data (Additional file 3: Figure S4). Moreover, in addition to previously observed C:G > A:T transversions, we observed C:G > G:C transversions to be caused by acoustic shearing of gDNA. Whilst the typical oxidative lesion product 8-oxo-G is established to directly induce C:G > A:T transversions, secondary oxidative lesion products of 8-oxo-G, including imidazolone, guanidinohydantoin, and spiroiminodihydantoin, may be responsible for causing C:G > G:C transversions [41, 44]. Thus, the oxidation of guanine residues may cause both C:G > A:T and C:G > G:C errors in response to the acoustic shearing of gDNA.

Recently, Chen et al. [45] showed that DNA damage accounts for the majority of erroneously identified variants reported to occur at frequencies ranging from 1–5% in public data sets, such as the 1000 Genomes Project and The Cancer Genome Atlas. Among these false negative results, the most prevalent substitution was C:G > A:T, followed by A:T > T:A. In contrast to Costello et al. [43], Chen et al. not only reported a moderate error frequency, but also asserted the limited effect of EDTA and/or nucleotide context specificity on DNA damage, indicating that the identified errors were not caused by reactive contaminants during purification. Chen et al. also demonstrated that observed C:G > A:T and A:T > T:A errors were virtually abolished by the use of a 1× TE (comprising 10 mM Tris (pH 8) and 1 mM EDTA) shearing buffer. Presumably, because we used the same 1× TE buffer for DNA shearing, the error rate in the present study was lower than that identified previously. In addition, the present study systematically analyzed errors (not limited to C:G > A:T transversions) that were found to persist after the removal of those errors induced by problematic and/or suboptimal conditions, which were discussed by previous studies. Thus, while previous studies significantly contributed to the identification and reduction of artifactual errors induced by DNA fragmentation, the present study provides additional valuable information that may enable the continued improvement of targeted deep sequencing methods.

The acoustic shearing condition we used in the present study was recommended by the manufacturer for fragmentation of input DNA with a median size of 150–200 bp. Whereas previous studies used DNA repair to specifically eliminate oxidative damage, we instead proposed a mild shearing condition to minimize the occurrence of such DNA damage in the first place. Given that our results show that the rate of C:G > A:T and C:G > G:C errors was attenuated by replacing the standard with mild shearing conditions, it may be beneficial to increase the input DNA fragment size to minimize induced errors. Moreover, since the maximum read length possible using the Illumina NGS platform has increased, and the produced base quality at the end of reads has improved, it may be more efficient to produce a greater data output in a single run by using longer DNA input fragments. On the other hand, a potential disadvantage of increasing DNA input fragment size is the broad distribution of fragment size, which may compromise the recovery rate of input DNA to a sequencing library. In addition, since DNA fragments longer than their corresponding target regions compromise the on-target rate, we observed that DNA fragmentation under the mild condition decreased the on-target capture rate by 15–25% compared to that achieved under the standard shearing condition. Thus, it may be more beneficial to produce a slightly greater amount of raw sequencing data than to compromise the depth of unique coverage when DNA fragmented under the mild condition is used as the input for capture-based targeted sequencing.

In the present study, we showed that the standard DNA shearing condition induced 8-oxo-G, which has been previously shown to mediate C:G > A:T and C:G > G:C errors. In addition to oxidative guanine, we also used an ELISA to evaluate apurinic-apyrimidinic (AP) sites in fragmented DNA, since depurination/depyrimidination represents one of the most common mechanisms underlying DNA damage [46]. We resultantly found that the identified AP sites significantly correlated with the ultrasonic acoustic energy level used during the shearing step (ANOVA, *p* value = 4.7× 10^−7^; Additional file 3: Figure S5b). Nevertheless, an additional DNA lesion-causing mechanism is likely active in this context, since these common lesions do not explain the A > G or A > T errors observed to occur at the end of DNA fragments. We therefore hypothesized that the increase in A > K errors at the end of fragments was likely associated with the mechano-chemical breakage of DNA. After we found the fragmentation-induced errors in our data, we analyzed additional paired PBL and plasma data sets (n = 3 for each) and consistently observed higher A > K error rates at the end of PBL DNA fragments than plasma DNA fragments (Additional file 3: Figure S12a). We further analyzed whole-exome sequence (WES) data sets from two independent studies that fragmented input DNA using acoustic shearing [47, 48]. We found the elevation of A > K errors at DNA break points in the WES data (Additional file 3: Figure S12b), but the degree of the increase was less prominent than that observed in our data. The quantitative difference might arise from variations in library construction conditions especially during end repair and adaptor ligation steps. For example, T4 DNA polymerase and Klenow fragment usually used for end repair have distinct characteristics including error rate; 1 × 10^−6^ bases for T4 DNA polymerase and 1 − 4 × 10^−4^ bases for Klenow fragment [49]. Both T4 DNA ligase and Taq ligase can be used for adaptor ligation, but display different specificities [50]. The differences in fidelity during end repair and specificity of DNA ligation might influence error rates at around DNA break points. Despite these variables, by comparing plasma and PBL DNA sequencing data generated under the same library construction condition, we were able to identify A > K error at the end of fragments associated with acoustic shearing. Nonetheless, the mechanism underlying the creation of A > K errors by acoustic shearing remains to be elucidated.

Notably, one insight into potential mechanisms is the preferential cleavage observed to occur at the 5′ phosphodiester bonds of A residues, which was consistent with previous studies [51, 52]. The cleavage preferences of PBL DNA fragmented by acoustic shearing were markedly different to those of nuclease-fragmented plasma DNA. We did not describe the pattern of plasma DNA fragmentation in detail in the present study, but we did note that the mono- and dinucleotide frequencies around cleavage sites in plasma DNA observed in the present study were consistent with those reported by previous studies (Additional file 3: Figures S9 and S11) [52, 53]. After we identified fragmentation-induced A > K substitutions to be localized at the end regions of the analyzed fragments in the present study, we analyzed sequence datasets from independent studies that each used acoustic shearing to fragment input DNA. Consistent with the results of the present study, we identified an elevated incidence of A > K errors at DNA break points in these additional sequence datasets (Additional file 3: Figure S12).

We estimated that after excluding errors induced by DNA fragmentation, the strand-specific hybrid selection step contributed to one-third of the remaining G > T and one-fifth of the remaining C > T errors. Our results were consistent with a previous report by Newman et al. [35] that also identified G > T and C > T errors to have been incurred during the hybrid selection step, although, using NimbleGen SeqCap as a capture reagent, Newman et al. reported a greater degree of G > T substitution than was observed in the present study. Since we estimated the errors incurred during the hybrid selection step by calculating the difference in error rates between complementary substitutions, it is possible that we may have underestimated the impact if there was C > A error on the minus strand during the hybrid selection step. Examining whole genome sequencing libraries generated using the same protocol, but without hybrid selection, would be a potential method to resolve this issue.

We next investigated whether biological background (i.e., intrinsic to the sample prior to isolation) significantly contributed to the error rate incurred during sequencing. Newman et al. [54] reported a marginally higher mean background rate to be present in plasma DNA “hotspot” variants compared to entire target regions, indicating that, in the absence of cancer, plasma DNA may carry somatic variants as a result of contributions from normal or pre-neoplastic cells. Since we observed that hotspot variants in our target regions did not evenly distribute across all substitution classes, and in fact biased toward classes with higher background rates (Additional file 3: Figure S13a), we selected data for a control group randomly, but proportionally with respect to the fractions of the various substitution classes observed in a given hotspot group. From this analysis, we found that the background rate of hotspots in both the plasma and PBL samples was not significantly different from that observed in the control group (Additional file 3: Figure S13b). We then assessed the background rate at tumor protein p53 (TP53) hotspots, which comprise approximately 29% of the total identified hotspots (52/189). TP53 is particularly relevant in this context, since TP53 variants are found in approximately half of solid tumors of varying cancer types, but occur less frequently in hematological malignancies [2, 55]. If plasma DNA carries TP53 variants as a result of contributions from pre-neoplastic cells of diverse tissues other than hematopoietic lineage cells, the error rate at TP53 hotspots in plasma samples should be elevated and different from that observed in PBL samples. We observed no difference in the background rate at TP53 hotspots between plasma and PBL DNA samples (Additional file 3: Figure S13c), implying the minimal impact of biological background at cancer hotspots. These data suggest that biological background was a minimal source of error during sequencing compared to that incurred as an artifact of various technical processes.

Notably, A:T > T:A and C:G > T:A transversion errors were slightly but significantly elevated in plasma DNA samples. It would be interesting to investigate whether this reflects a specific set of errors induced by the release of genomic DNA into the blood to form cell-free DNA (cfDNA), by the events that trigger this release, or by DNA damage incurred during the circulation of cfDNA in plasma. Further research is required to establish whether plasma DNA contains more biological background errors than PBL DNA prior to isolation. This would in turn reveal whether differing DNA isolation techniques between plasma and cells creates technical background errors.

Given that background errors are not entirely random, and in fact display significant site-specific variations, site-specific error rate distributions generated from real sequencing data have been used to distinguish somatic variants from background errors. For example, both plasma and PBL DNA samples have been used as a control group to detect circulating tumor DNA (ctDNA). However, the level of similarity between background errors in plasma versus PBL DNA samples is not established. Our results pertaining to the background rates across substitution classes showed similar patterns depending on sequence contexts between plasma and PBL DNA samples. However, some significant differences suggested that plasma samples from healthy donors would be suitable for use as control samples for the detection of ctDNA. If PBL samples are used as an alternative resource to estimate the site-specific error rate distributions of plasma DNA in normal controls for some practical reason, our results suggest that it should be fragmented under a mild shearing condition to minimize incurred errors.

## Conclusions

Despite extremely low mean error rates, baseline noise during sequencing can drastically compromise the specific detection of low-allelic fraction variants due to stochastic and site-specific variations in errors, which may be mitigated by increasing the depth of coverage. In the present study, we comprehensively analyzed errors embedded in targeted deep sequencing data to uncover their characteristics and identify potential causes for background noise. Our results provide information on error patterns and their causes that may be invaluable in elucidating the limitations of, and thus improving the use of, targeted deep sequencing methods to detect low-allelic fraction variants.

## Methods

### Plasma and PBL sample collection

Blood samples were collected in Cell-Free DNA™ BCT tubes (Streck Inc., Omaha, NE, USA) [56] from two healthy adults and 17 patients with pancreatic cancer (Additional file 2: Table S5). Blood samples were processed within 6 h of collection via three graded centrifugation steps (840 g for 10 min, 1040 g for 10 min, and 5000 g for 10 min, at 25 °C). PBLs were drawn from the initial centrifugation, and plasma was transferred to new microcentrifuge tubes at each step. Plasma and PBL samples were stored at −80 °C until cfDNA extraction.

### DNA extraction

Germline DNAs from collected peripheral blood mononuclear cells were isolated using a QIAamp DNA mini kit (Qiagen, Santa Clarita, CA, USA). Circulating DNAs were extracted from 1–5 mL of plasma using a QIAamp Circulating Nucleic Acid Kit (Qiagen). DNA concentration and purity were assessed by a PicoGreen fluorescence assay using a Qubit 2.0 Fluorometer (Life Technologies, Grand Island, NY, USA) with a Qubit dsDNA HS Assay Kit and a BR Assay Kit (Thermo Fisher Scientific, Waltham, MA, USA). DNA concentration and purity were quantified using a Nanodrop 8000 UV-Vis spectrometer (Thermo Fisher Scientific) and a Picogreen fluorescence assay using a Qubit 2.0 Fluorometer (Life Technologies). The fragment size distribution was measured using a 2200 TapeStation Instrument (Agilent Technologies, Santa Clara, CA, USA) and real-time PCR Mx3005p (Agilent Technologies) according to the manufacturer’s instructions.

### Library preparation

Genomic DNAs from PBL samples were fragmented to 150 − 200 bp using a Covaris S220 (6 min, 10% duty factor, peak incident power = 175 W, 200 cycles/burst; Covaris Inc., Woburn, MA, USA). Plasma DNA was prepared without fragmentation. The construction of sequencing libraries was achieved using 200 ng (for all samples) of PBL and 37.3 ng (on average) of plasma DNA. To test whether DNA fragmentation influenced the background error rate, the intensity and/or duration of DNA fragmentation was varied using 200 ng of initial genomic DNA from HapMap samples (Additional file 2: Table S4). The libraries for PBL and plasma DNAs were constructed using a KAPA Hyper Prep Kit (Kapa Biosystems, Woburn, MA, USA) as described previously [30]. Briefly, end repair, A-tailing, adapter ligation, and PCR reactions (nine amplification cycles) prior to target enrichment were performed according to the manufacturer’s recommended protocols. A purification step was carried out using AMPure beads (Beckman Coulter, Indiana, USA) after each step. Adaptor ligation was performed using a pre-indexed PentAdapter™ (PentaBase ApS, Denmark) at 4 °C overnight.

### Target enrichment and sequencing for liquid biopsies

We designed unique RNA baits to target ~499 kb of the human genome, including exons from 83 cancer-related genes. Up to eight purified libraries were pooled and adjusted to a total of 750 ng for each hybrid selection reaction. Target enrichment was performed following the SureSelect bait hybridization protocol with the modification of replacing the blocking oligonucleotide with IDT xGen blocking oligonucleotide (IDT, Santa Clara, CA, USA) for the pre-indexed adapter. After the target enrichment step, the captured DNA fragments were amplified via 13 PCR cycles using P5 and P7 oligonucleotides. The amplified library was purified with AMPure beads and quantified by Picogreen fluorescence assay using a Qubit 2.0 Fluorometer (Life Technologies) with a dsDNA HS Assay Kit (Thermo Fisher Scientific). The size distribution was analyzed using a 2100 Bioanalyzer (Agilent Technologies). Based on DNA concentration and average fragment size, the libraries were normalized to an equal concentration of 2 nM and pooled by equal volume. After denaturing using 0.2 N NaOH, the libraries were diluted to 20 pM with a hybridization buffer (Illumina, San Diego, CA, USA). Cluster amplification of denatured templates was performed according to the manufacturer’s protocol (Illumina). Flow cells were sequenced in the 100-bp paired-end mode using the HiSeq 2500 v3 Sequencing-by-Synthesis Kits (Illumina) and then analyzed using RTA software (v.1.12.4.2 or later).

### Sequence data processing

Using BWA-mem (v0.7.5) [57], all raw data were aligned to the hg19 human reference to create BAM files. SAMTOOLS (v0.1.18) [58], Picard (v1.93), and GATK (v3.1.1) [59] were used for sorting SAM/BAM files, local realignments, and duplicate markings, respectively. Through the process, we filtered reads to remove duplicates, discordant pairs, and off-target reads.

### Background allele

For each paired set of PBL and plasma DNA samples, we determined a base at a position across the entire target regions to be a background allele if the following conditions were met: (1) the base was a non-reference allele; (2) the position displayed sufficient depth of coverage (i.e., >500×) in the paired PBL and plasma DNA samples; and (3) the frequencies of the base in both samples did not indicate a germline variant (i.e., <5%). Since we used samples from cancer patients, we also removed the candidate alleles for somatic cancer variants. This was achieved by generating sequencing data for matched fine-needle aspiration (FNA) biopsies obtained from patients with cancer at a time close to that of blood collection, prior to therapeutic treatments. For example, *KRAS* variants (Additional file 2: Table S5) were removed from the analysis if detected in the matched FNA specimens. Sequencing libraries for the primary tumors were generated using 200 ng of input DNA and sequenced on the HiSeq2500 as described above. The depth of coverage of sample DNA after removal of duplication in FNA samples was on average 987.15× (790.32 − 1476.55×). In a given paired set of PBL and plasma samples, we excluded a position if the depth of coverage at that position was below 250× in the matched FNA biopsy and an allele if it was present at a frequency greater than 2.5% in the FNA sample.

### Analysis of nucleotide composition and substitution rate in the vicinity of a DNA break point

To evaluate the frequency of mono- and dinucleotides at positions around a DNA break point, the 5′ end position of each mapped read was determined by alignment to the human reference genome, and the sequence for the region of 100 bp (±50 bp) around the DNA break point was then obtained. For consistency, the collected sequences were displayed in the direction of the positive strand of the reference genome. To accurately determine the genomic positions of fragment ends, fragments that displayed any clipping at their terminal section were excluded. Alignment data (BAM or SAM) were analyzed via a Python (v2.7.6) script that we constructed using the Pysam (v0.9.1.4) library. The frequencies of nucleotides were calculated as the number of occurrences of a given mono-/dinucleotide divided by the total number of bases with a quality score ≥30 at positions relative to the DNA break point. The frequencies were calculated for each sample, and these frequency values from the 19 samples were then averaged. To estimation the frequency of mononucleotides, we determined the position as the number of nucleotides from the first 5′ end nucleotide of the read. For dinucleotides, the number of nucleotides between the phosphodiester bond in a given dinucleotide and the break point was used to indicate the position relative to the DNA break point. For instance, “distance zero” indicated that the first nucleotide occurred immediately before the 5′ end of the read, and the second nucleotide coincided with the beginning of the read.

Background error rates across all substitution classes were also calculated at each position relative to the DNA break point. The background alleles for each sample (defined as described in the previous section) were used for this analysis. For a comparison between PBL and plasma DNA samples, the substitution rate was normalized against the average rate of 1 − 50 bp. To remove errors incurred in the Illumina sequencing, we used only R1 reads whose initial sections showed relatively higher quality scores than those of R2 reads.

### Quantification of 8-oxo-G and AP sites by ELISA

The levels of 8-oxo-G in the samples were assessed using an HT 8-oxo-dG ELISA Kit II (Trevigen, Gaithersburg, MD, USA) according to the manufacturer’s instructions. Fragmented DNA from each sample (500 ng) was used as the input for all ELISAs. After optical density measurements were taken at 450 nm using a microplate absorbance reader, concentrations were calculated on the basis of a linear calibration curve generated for each experiment using 8-oxo-G standard solutions. AP sites were quantified using the OxiSelect™ Oxidative DNA Damage Quantitation Kit (Cell biolabs, San Diego, CA, USA) according to the manufacturer’s instructions. Briefly, 500 ng of genomic DNA was labeled with an aldehyde-reactive probe (ARP) containing a biotin moiety, before being immobilized in a micro-well and incubated with streptavidin-conjugated HRP. The DNA was next incubated with an HRP substrate and the resulting absorbance measured at 450 nm. To infer the number of AP sites from the measured absorbance units, a standard curve was generated using serial dilutions of a stock standard solution containing 40 ARP/105-bp of DNA.

## Additional files

Additional file 1: Table S1. Findings and methods from studies charactering artifactual substitutions in sequencing data. (DOCX 29 kb)

Additional file 2: Table S2. Genes targeted for massively parallel sequencing. **Table S3.** Summary of sequencing metrics for 19 paired plasma and PBL DNA samples. **Table S4.** Acoustic shearing conditions. **Table S5.** Donor characteristics and KRAS variant allele frequency. (XLSX 19 kb)

Additional file 3: Figure S1. Single nucleotide polymorphism concordance between plasma and PBL DNA samples. **Figure S2.** The distribution of base quality scores from generated sequencing data. **Figure S3.** The proportion of consistent/inconsistent errors between paired reads 1 and 2. **Figure S4.** Background allele frequencies of 12 substitution classes. **Figure S5.** Mutagenic DNA lesions are associated with high DNA-shearing conditions. **Figure S6.** Background allele frequencies around the DNA break point. **Figure S7.** Normalized mononucleotide frequencies around the DNA break point. **Figure S8.** Dinucleotide frequencies around the DNA break point. **Figure S9.** Comparison of dinucleotide frequencies around the DNA break point between PBL and plasma DNA samples. **Figure S10.** Density plot of allelic background rate. **Figure S11.** Mononucleotide frequencies of plasma DNA fragments around the DNA break point. **Figure S12.** Analysis of the DNA break point. **Figure S13.** Analysis of background allele frequency in “hotspot” mutations. (PDF 1649 kb)

## Abbreviations

8-oxo-G: 8-oxo-7,8-dihydroguanine
AP site: Apurinic-apyrimidinic site
cfDNA: Cell-free DNA
ctDNA: Circulating tumor DNA
ELISA: Enzyme-linked immunosorbent assay
FNA: Fine needle aspiration
gDNA: Genomic DNA
NSG: Next-generation sequencing
PBL: Peripheral blood leukocyte
SD: Standard deviation
SNP: Single nucleotide polymorphism
SNV: Single nucleotide variant
