# Supplementary material for: Characterization of background noise in capture-based targeted sequencing data
Source: Genome Biol. 2017 Jul 21;18:136. doi: 10.1186/s13059-017-1275-2 (PMC5521083; doi:10.1186/s13059-017-1275-2)
Supplement: Supplementary file 1 — Findings and methods from studies charactering artifactual substitutions in sequencing data. (DOCX 29 kb) [file 13059_2017_1275_MOESM1_ESM.docx]

**Table S1. Findings and methods from studies charactering artifactual substitutions in sequencing data.**

In each study, the affected substitution classes were listed in decreasing order of error frequency. The errors due to DNA damage during the hybrid capture step were described with respect to the plus strand of the reference genome, which were different between Newman et al. and Park et al because of the difference in the strand specificity of hybrid capture baits. A→K* due to acoustic shearing was observed only around DNA break points. *Abbreviations:* *WES* whole exome sequencing, *WGS* whole genome sequencing, *TDS* targeted deep sequencing, *PBL* peripheral blood leukocyte, *8-oxo-G* 8-oxo-7,8-dihydroguanine

| Study | Primary cause of the errors | Affected substitution class | Error frequency | Primary method to estimate the level of errors | Base quality score | Type(s) of data | Controlled experiments | | |
| --- | --- | --- | --- | --- | --- | --- | --- | --- | --- |
|  |  |  |  |  |  |  | Variable(s) | Measurement | Experimental conditions |
| Costello *et al*. [[1](#_ENREF_1)] | Acoustic shearing in samples containing reactive contaminants from the extraction process | C:G→A:T | <20% | ArtQ: -10×log10(consistent errors-inconsistent errors/all observation) “consistent” means matching with the identified artifact characteristics | >Q20 | WES  WGS | Shearing condition; shearing intensity and shearing solution | ArtQ and 8-oxo-G level | Shearing : Covaris E210,  Library construction : Agilent SureSelectXT Hybrid selection : Agilent SureSelectXT Sequencing run : HiSeq2000 |
| Chen *et al*.[[2](#_ENREF_2)] | Mutagenic DNA damage due to acoustic shearing | C:G→A:T  A:T→T:A | ~ 1 − 5 % | Variant frequency and GIV-score (an index value indicating imbalance of variant numbers of a substitution class between R1 and R2) | >Q30 | WES  WGS  TDS | Shearing condition; buffer concentrations and in vitro DNA repair | Variant frequency | Shearing : Covaris S2 Library construction : NEBNext Ultra II  Hybrid selection : Agilent's ClearSeq comprehensive cancer panel  Sequencing run : MiSeq |
| Newman *et al*. [[3](#_ENREF_3)] | DNA damage during the hybrid capture step | G→T C→T | << 1% | Imbalance of error frequency between complimentary substitution classes | >Q30 | TDS | Hybridization time (from 0.1 to 3days) | Ratio of error frequencies | Shearing : No (plasma DNA) Library construction : KAPA  Hybrid selection : a custom NimbleGen SeqCap EZ Choice  Sequencing run : Illumina sequencers |
| Park *et al*. [this study] | Sequencing run | across all substitution classes | << 1% | Concordance between R1 and R2 | >Q30 | TDS | − | − | Shearing : Covaris S220 Library construction : KAPA Hybrid selection : a custom Agilent SureSelectXT  Sequencing run : HiSeq2500 |
|  | Mutagenic DNA damage due to acoustic shearing | C:G→A:T C:G→G:C A→K* |  | Error frequency difference between PBL and plasma DNA samples |  |  | Shearing intensity | Error frequency and 8-oxo-G level |  |
|  | DNA damage during the hybrid capture step | C→A G→A |  | Imbalance of error frequency between complimentary substitution classes |  |  | − | − |  |

**References**

1. Costello M, Pugh TJ, Fennell TJ, Stewart C, Lichtenstein L, Meldrim JC, Fostel JL, Friedrich DC, Perrin D, Dionne D, et al: **Discovery and characterization of artifactual mutations in deep coverage targeted capture sequencing data due to oxidative DNA damage during sample preparation.** *Nucleic Acids Res* 2013, **41:**e67.

2. Chen L, Liu P, Evans TC, Jr., Ettwiller LM: **DNA damage is a pervasive cause of sequencing errors, directly confounding variant identification.** *Science* 2017, **355:**752-756.

3. Newman AM, Lovejoy AF, Klass DM, Kurtz DM, Chabon JJ, Scherer F, Stehr H, Liu CL, Bratman SV, Say C, et al: **Integrated digital error suppression for improved detection of circulating tumor DNA.** *Nat Biotechnol* 2016, **34:**547-555.
