# Supplementary material for: Characterization of background noise in capture-based targeted sequencing data
Source: Genome Biol. 2017 Jul 21;18:136. doi: 10.1186/s13059-017-1275-2 (PMC5521083; doi:10.1186/s13059-017-1275-2)
Supplement: Supplementary file 3 — Single nucleotide polymorphism concordance between plasma and PBL DNA samples. Figure S2. The distribution of base quality scores from generated sequencing data. Figure S3. The proportion of consistent/inconsistent errors between paired reads 1 and 2. Figure S4. Background allele frequencies of 12 substitution classes. Figure S5. Mutagenic DNA lesions are associated with high DNA-shearing conditions. Figure S6. Background allele frequencies around the DNA break point. Figure S7. Normalized mononucleotide frequencies around the DNA break point. Figure S8. Dinucleotide frequencies around the DNA break point. Figure S9. Comparison of dinucleotide frequencies around the DNA break point between PBL and plasma DNA samples. Figure S10. Density plot of allelic background rate. Figure S11. Mononucleotide frequencies of plasma DNA fragments around the DNA break point. Figure S12. Analysis of the DNA break point. Figure S13. Analysis of background allele frequency in “hotspot” mutations. (PDF 1649 kb) [file 13059_2017_1275_MOESM3_ESM.pdf]

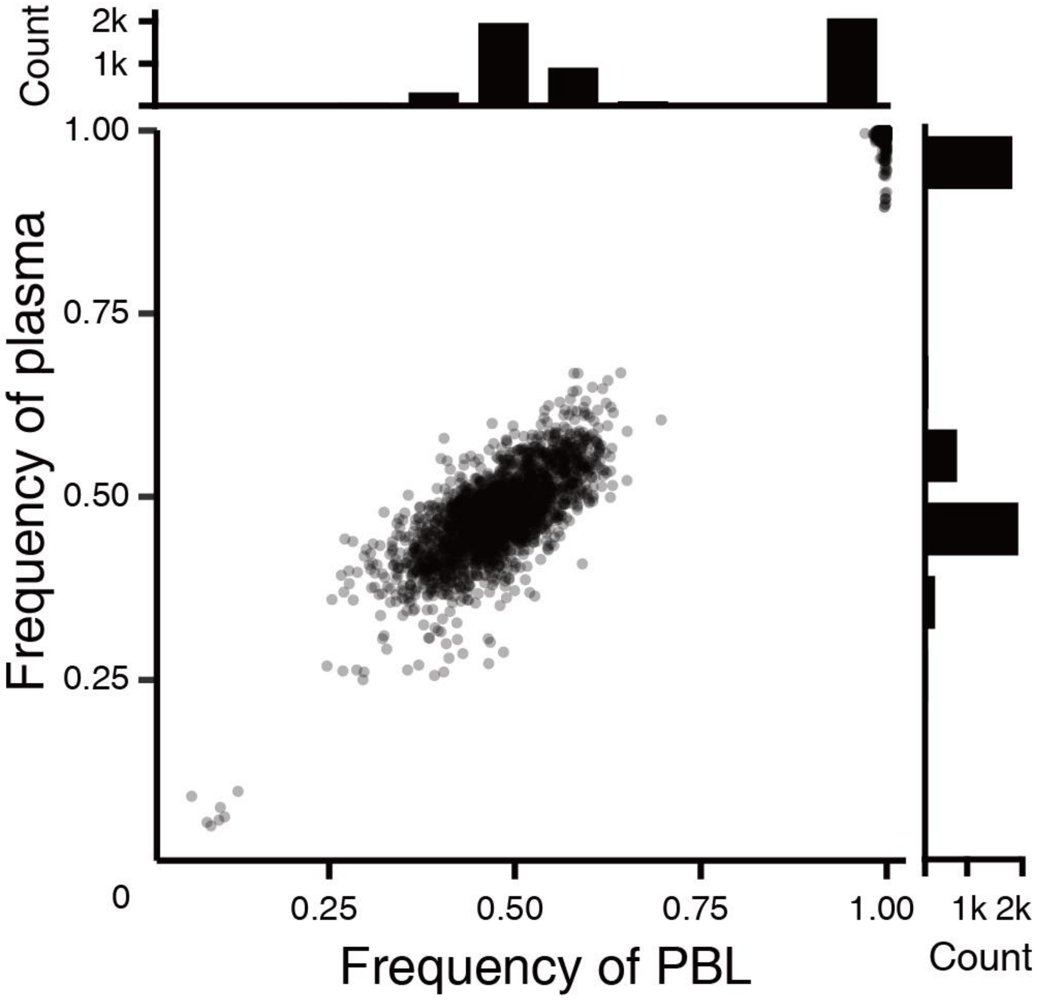

Figure S1. Single nucleotide polymorphism concordance between plasma and PBL DNA samples. Non-reference alleles present at an allele frequency greater than 5% in at least one of the paired plasma and/or PBL samples were selected for analysis. In total the allele frequencies of 5,133 SNPs (from 19 sample pairs) in plasma (*y*-axis) and PBL samples (*x*-axis) were plotted. SNP allele frequencies in plasma and PBL DNA samples were found to be strongly correlated (R = 0.9913, p-value < 0.0001).

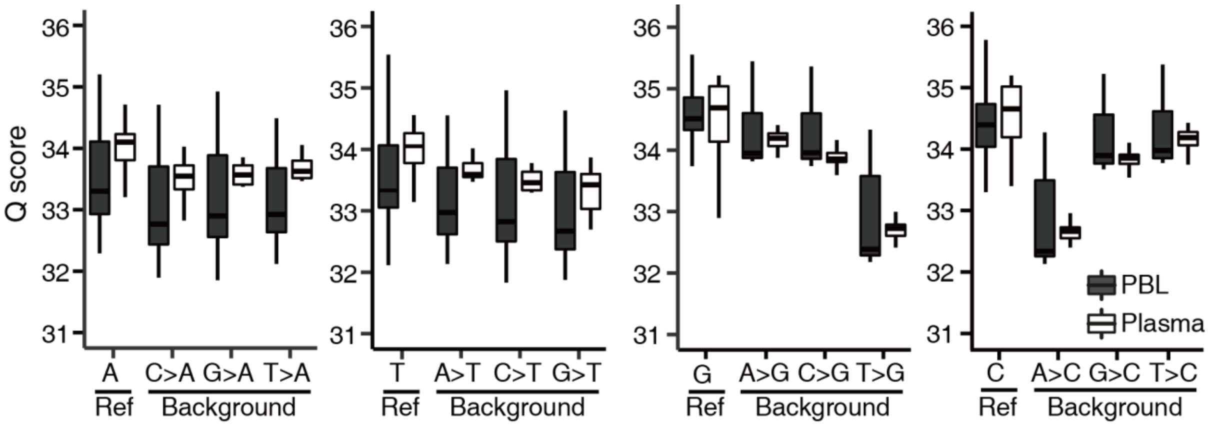

Figure S2. The distribution of base quality scores from generated sequencing data. For each of the four nucleotides after deduplication and filtration of bases with a quality score < 30, base quality scores were compared between reference and background alleles. The background alleles of each nucleotide were divided into three groups dependent on the reference base.

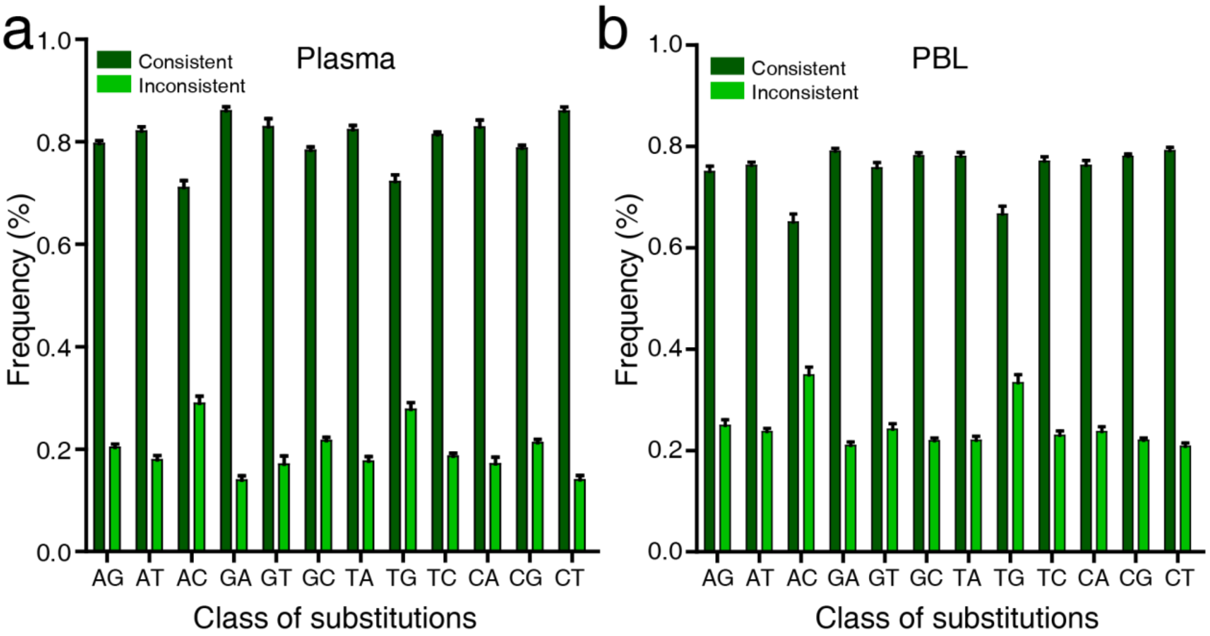

Figure S3. The proportion of consistent/inconsistent errors between paired reads 1 and 2. The average error fractions were calculated from plasma **(a)** and PBL **(b)** DNA samples. Error bars represent the s.e.m.

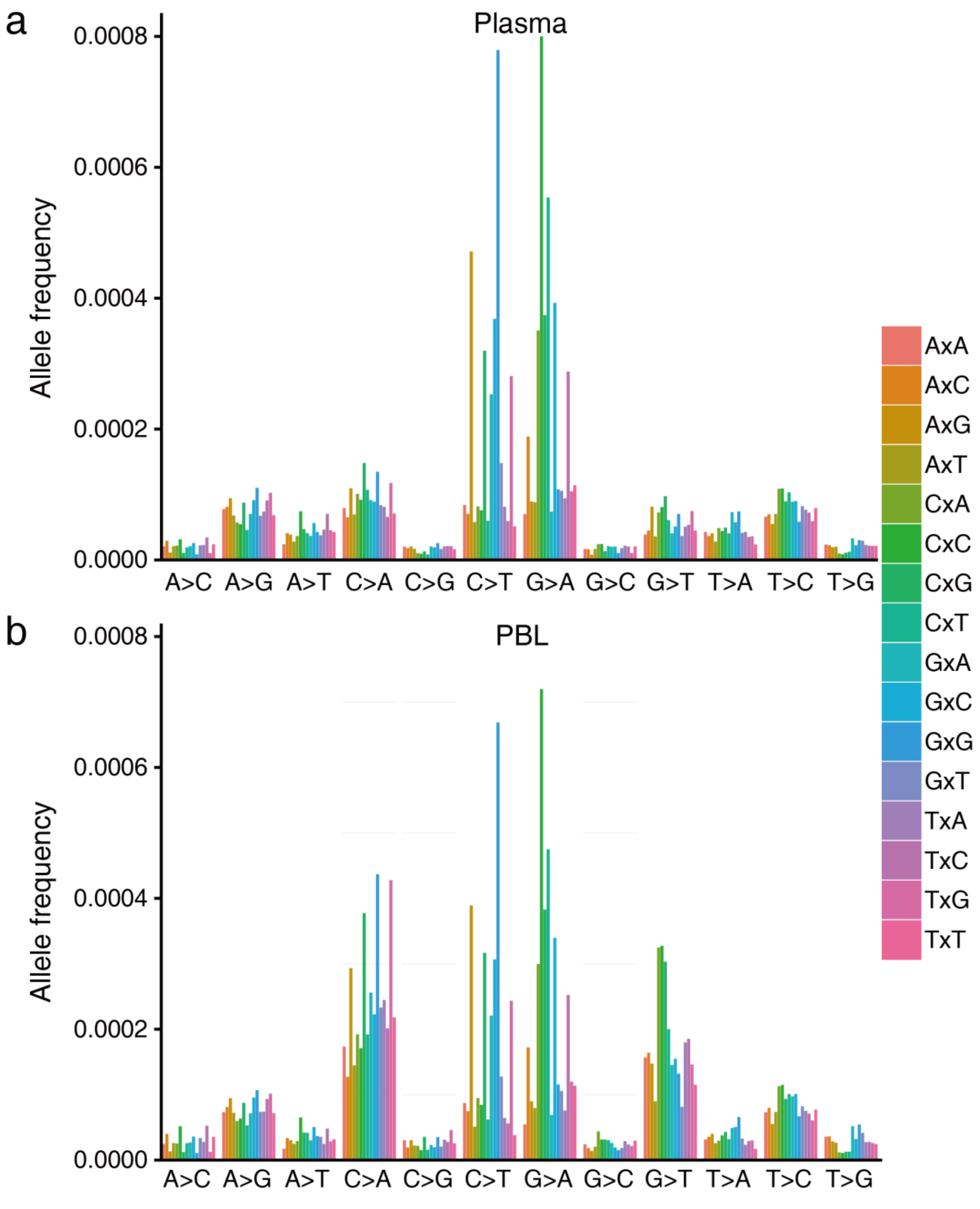

Figure S4. Background allele frequencies of 12 substitution classes. The sequence context-dependent background allele frequencies were estimated in plasma **(a)** and PBL **(b)** DNA samples. The substitution types were denoted on the x-axis, and the 4×4 combinations of possible preceding (5') or trailing (3') bases are color-coded as indicated in the legend on the right.

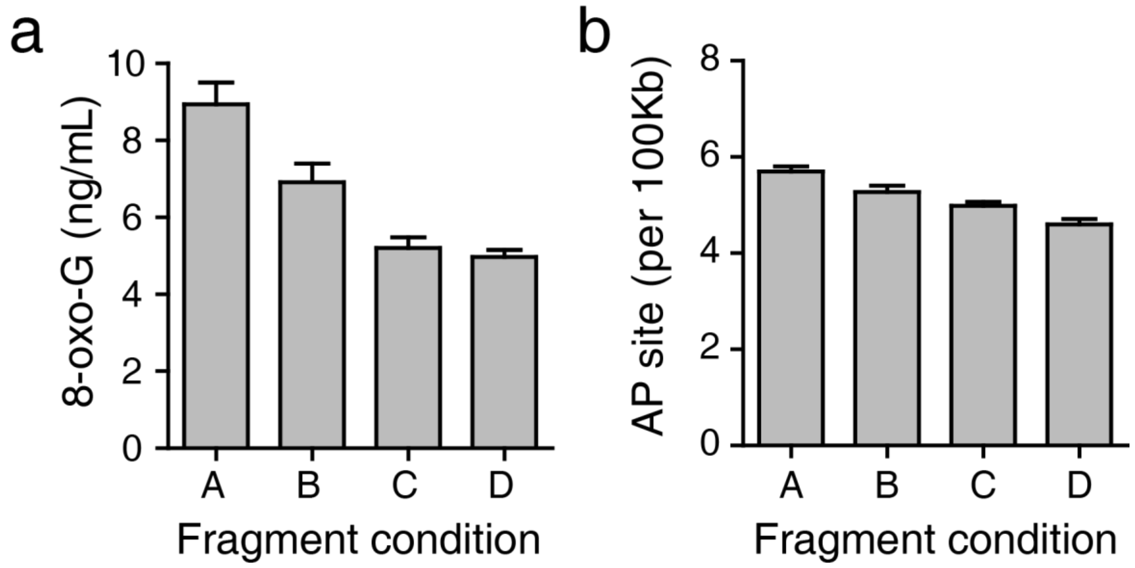

Figure S5. Mutagenic DNA lesions are associated with high DNA-shearing conditions. The results of the conducted ELISA evaluating **(a)** 8-oxo-G and **(b)** apurinic-apyrimidinic (AP) sites in cell-line genomic DNA samples (n = 9) fragmented under different shearing conditions. The specific parameters for each condition are described in Figure 3 and Additional file 1: Table S3. All other conditions were significantly different to the standard condition (A) (ANOVA, *p*-value < 0.001). Error bars represent the s.e.m.

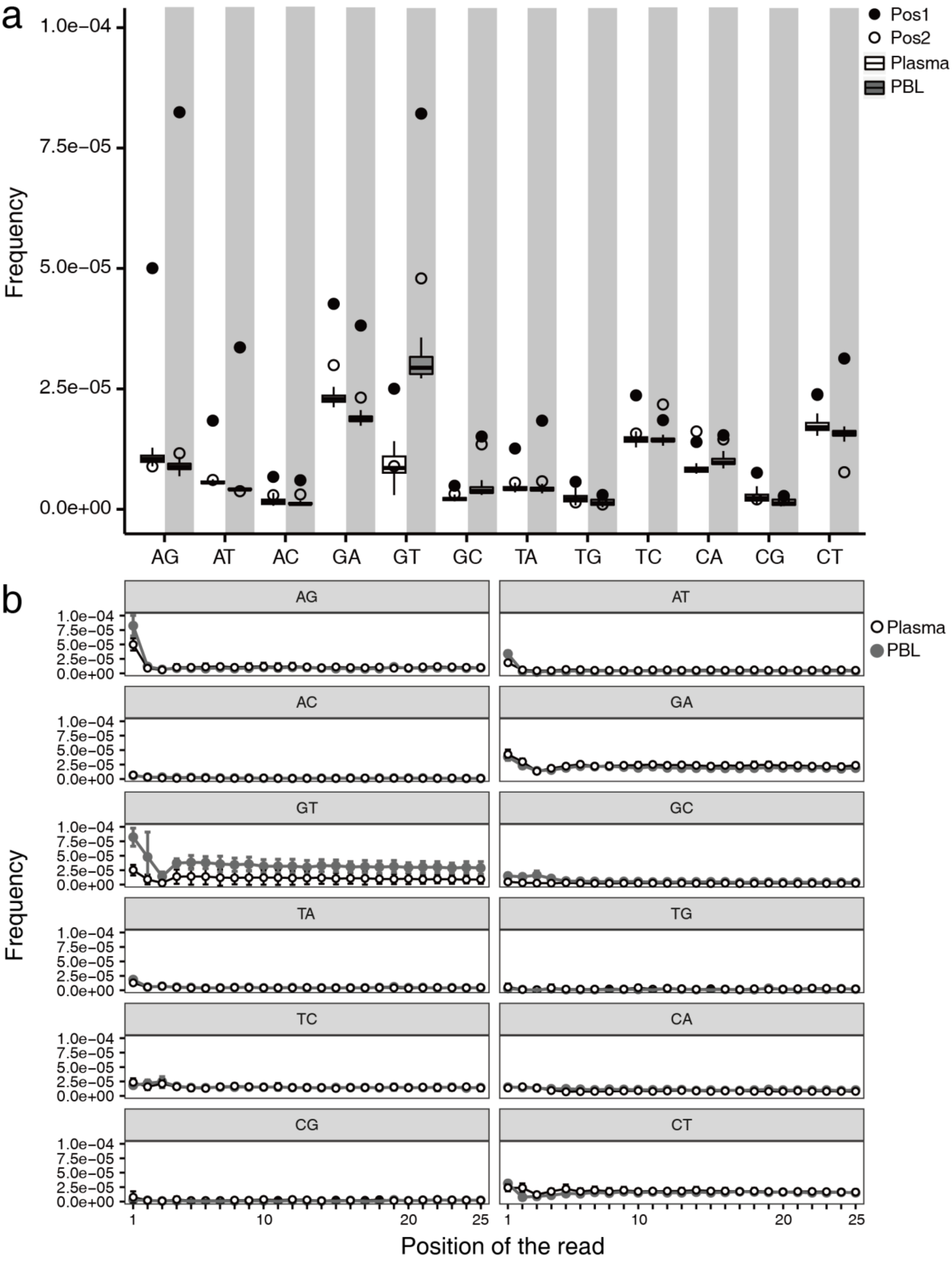

Figure S6. Background allele frequencies around the DNA break point. (a) The distribution of frequencies in 1–50 bp was box-plotted for each substitution class. On top of each box plot, the allele frequencies at the first and second bases were displayed for comparison. **(b)** Background allele

frequencies for each substitution class were calculated for each DNA sample. The average frequency (calculated from the 19 samples) for each class is plotted against the nucleotide position of the read (x-axis). Error bars indicate standard deviation.

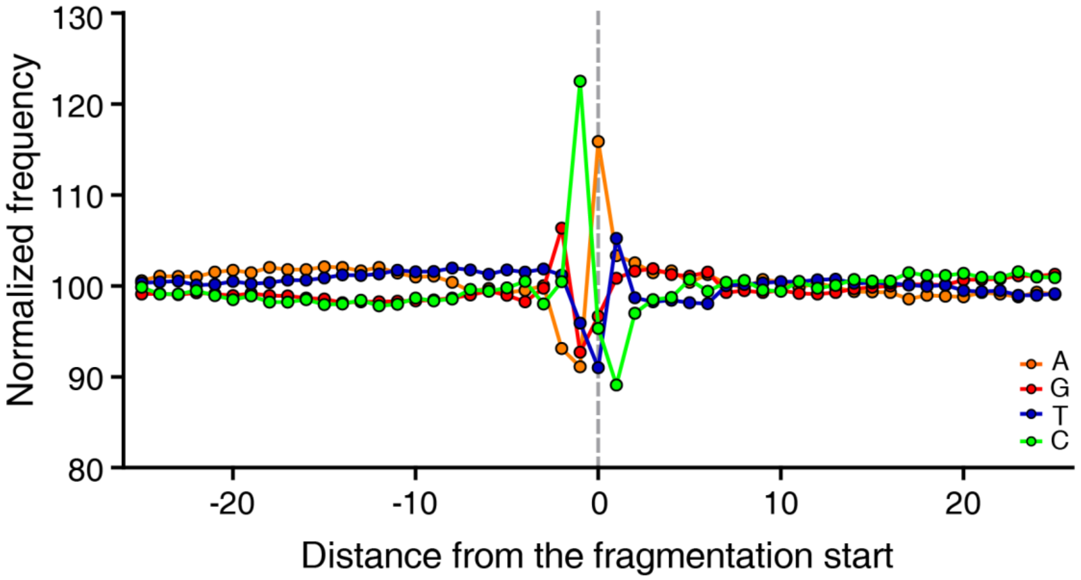

Figure S7. Normalized mononucleotide frequencies around the DNA break point. The normalized frequency for each nucleotide was calculated by dividing the frequency at each position by the average frequency across the region of 50 bp (i.e. from –25 to +25).

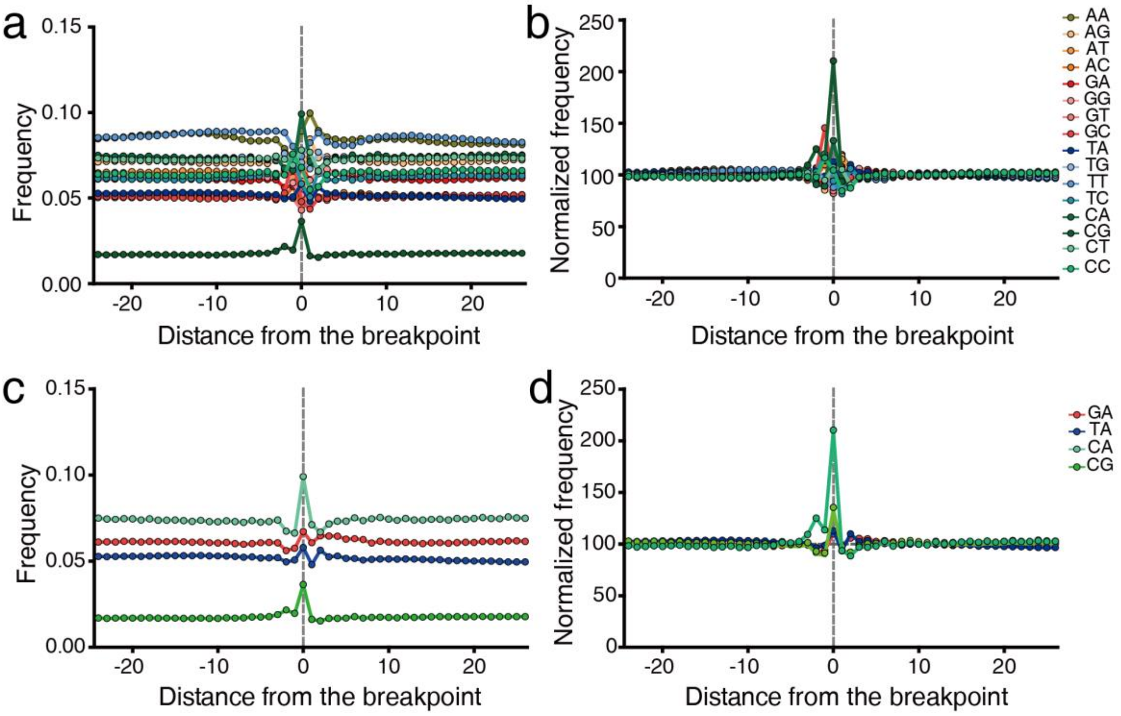

Figure S8. Dinucleotide frequencies around the DNA break point. For each of 16 dinucleotides, frequencies **(a),** and normalized frequencies **(b),** were plotted against position relative to the DNA break point (x-axis). Based on normalized frequencies at the zero position, the four dinucleotides that displayed a high level of enrichment at the DNA break point were selected. For each of these four dinucleotides, frequencies **(c),** and normalized frequencies **(d),** were displayed.

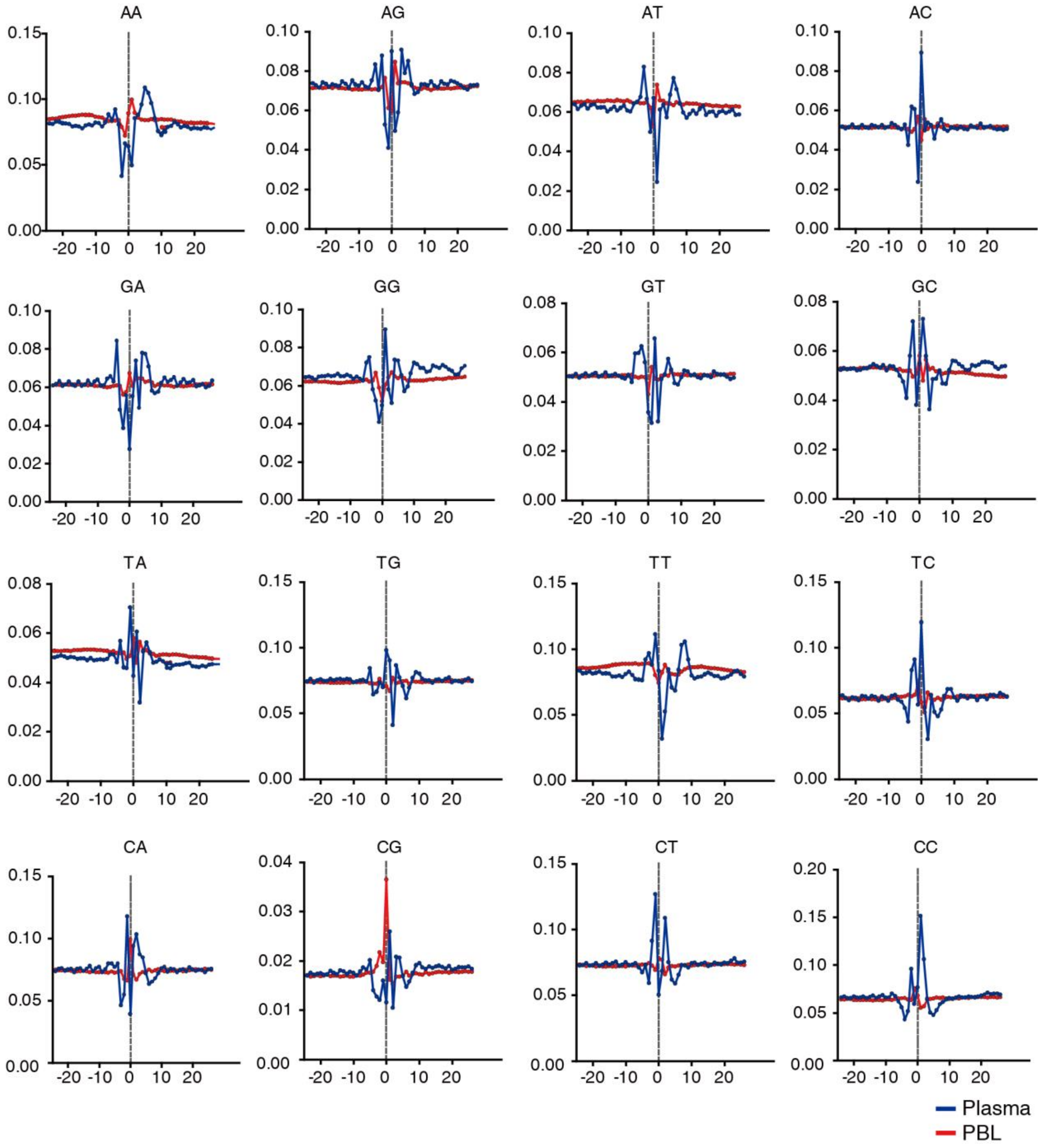

Figure S9. Comparison of dinucleotide frequencies around the DNA break point between PBL and plasma DNA samples.

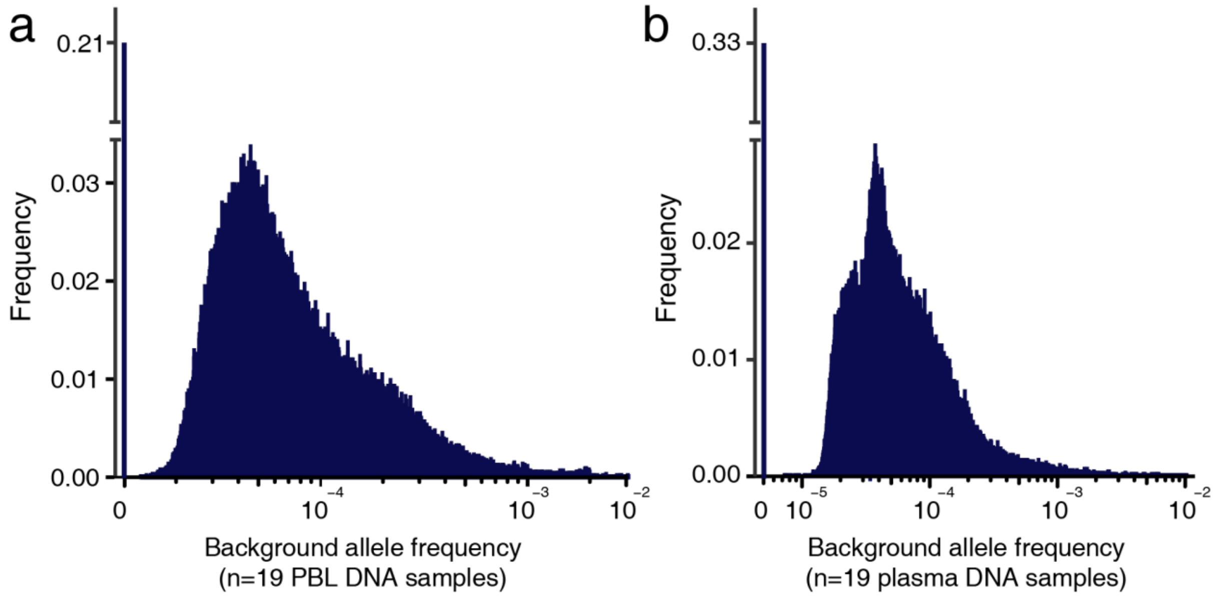

Figure S10. Density plot of allelic background rate. For each background allele across the entire target regions, the average background allele frequency was calculated from 19 pairs of PBL **(a)** and plasma **(b)** DNA samples. Density was calculated after removing error-free alleles from all 19 DNA samples. The allele frequency of error-free alleles was 21.5% for PBL, and 33.2% for plasma DNA samples, respectively.

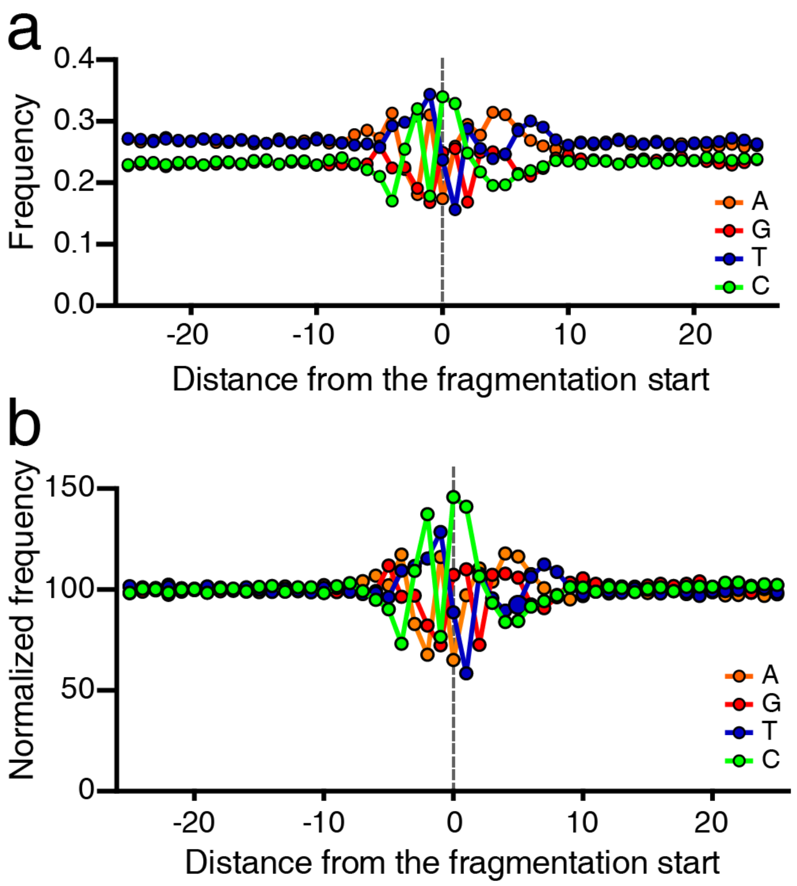

Figure S11. Mononucleotide frequencies of plasma DNA fragments around the DNA break point. For four mononucleotides, frequencies **(a),** and normalized frequencies **(b),** are plotted against position relative to the DNA break point (x-axis).The normalized frequency for each nucleotide was calculated by dividing the frequency at each position by the average frequency across the region of 50 bp (from –25 to +25).

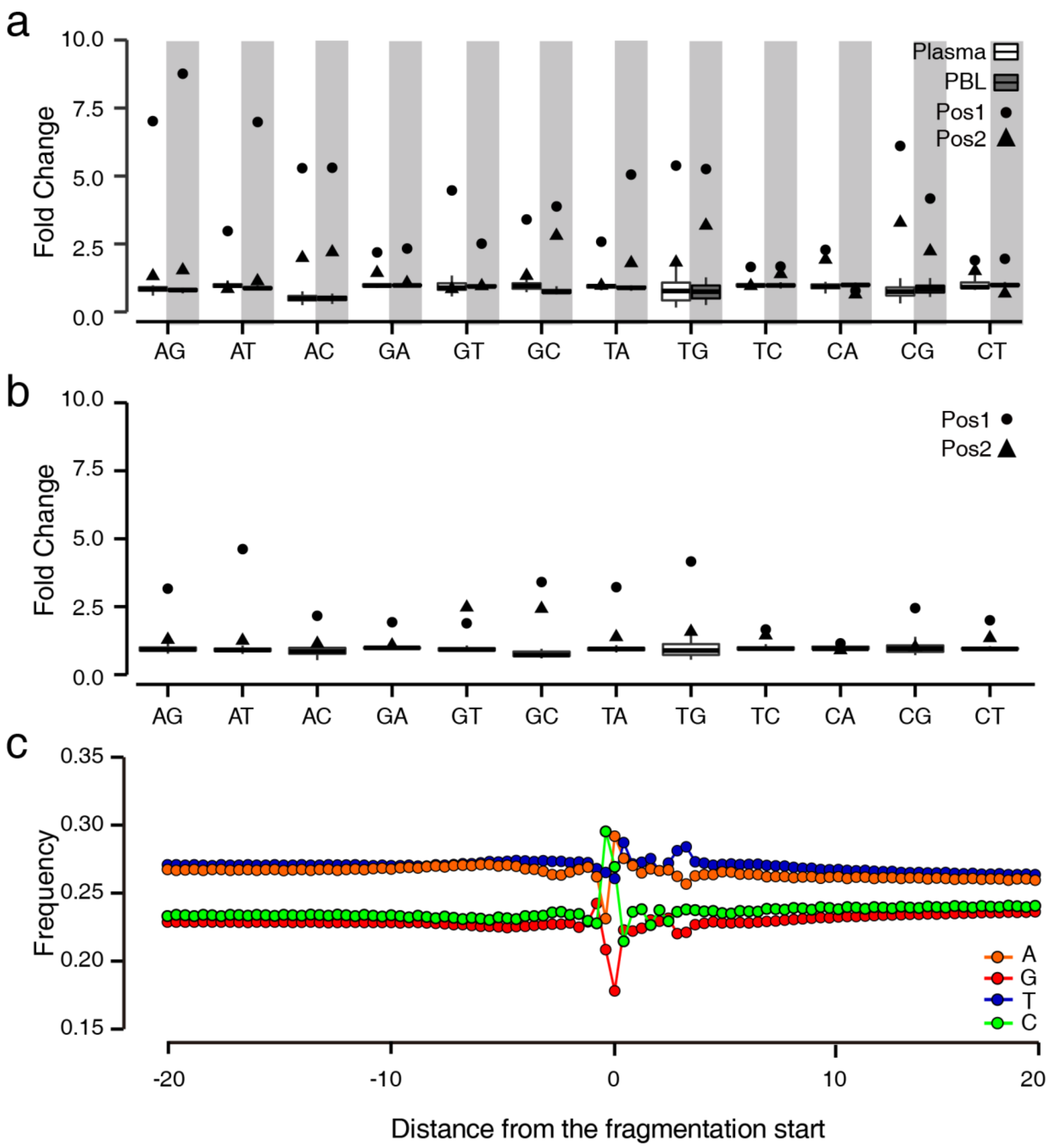

Figure S12. Analysis of the DNA break point. (a, b) Fold changes in error rates were calculated by dividing the substitution rate at a given position by the average rate of 1–50 bp. The distribution of fold changes across 1–50 bp is shown (box plots), and the observed fold change at the first and the second bases is marked (above box plots) for comparison. **(a)** Additional plasma and PBL DNA sample data (n = 3) and **(b)** whole-exome sequencing (WES) data from two independent studies were analyzed. The WES data with the following accession numbers were analyzed: SRR309291, SRR309292, SRR309293 (from the NCBI Sequence Read Archive); ERR850376 (from the European Nucleotide Archive). **(c)** Mononucleotide frequencies around the DNA break point using the WES data.

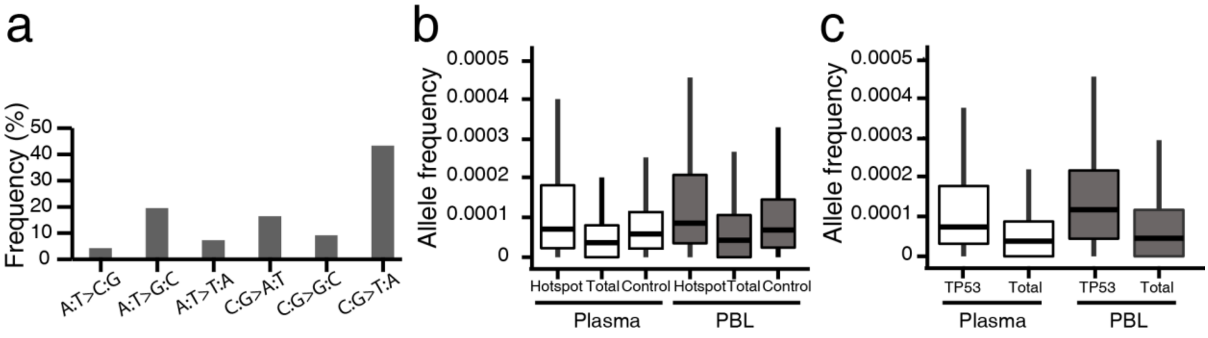

Figure S13. Analysis of background allele frequency in 'hotspot' mutations. (a) The fraction of each substitution class in hotspot mutations was plotted. **(b, c)** The average background allele frequencies for the 19 DNA samples were box-plotted. **(b)** The average frequency of background alleles in hotspot mutations, entire target regions, and a control group was calculated for each sample. The control group was generated by randomly selecting background alleles according to the substitution distribution in hotspot mutations. **(c)** The frequencies of hotspot and total background alleles in TP53 were compared between PBL and plasma DNA samples.
